# Editorial: Amaranthus: naturally stress-resistant resources for improved agriculture and human health, volume II

**DOI:** 10.3389/fpls.2023.1329377

**Published:** 2023-12-13

**Authors:** Matthew W. Blair, Chance Riggins, Ana Paulina Barba de la Rosa

**Affiliations:** ^1^ Department of Agricultural and Environmental Sciences, Tennessee State University, Nashville, TN, United States; ^2^ Department of Crop Sciences, University of Illinois at Urbana-Champaign, Champaign, IL, United States; ^3^ Molecular Biology Division, Instituto Potosino de Investigación Científica y Tecnológica (IPICYT), San Luis Potosí, Mexico

**Keywords:** grain amaranth, vegetable amaranth, abiotic stress, C4 photosynthesis, diversified agriculture, new crop, weed relatives

In this edition of the Research Topic, we discuss advantages of the genus *Amaranthus*, which is a group of dicotyledonous plants with adaptation traits to abiotic and climate stresses. Some are used as sources of grain, flours, leafy vegetables, fodders and industrial products. As a C4 photosynthetic group, *Amaranthus* have more efficient carbon fixation than C3 photosynthetic plants especially at high temperatures and under drought (Das, 2016). As carbon dioxide in the atmosphere has increased some of this advantage may diminish but their drought, salt and heat tolerance strategies are valuable (see articles discussed in previous Research Topic issues editorial of Riggins et al. (2021).

For climate change specifically, water-use efficiency and tolerance to water deficit with high temperatures (Vargas-Ortiz et al., 2021) provide adaptation of amaranth species to a warming world. This is based on biochemical changes as shown in this Research Topic by Reyes-Rosales et al. According to these latter authors, scenarios of constant heat stress may lead to reproductive failure of grain amaranths but leaf retention and recovery from heat shock treatments are valuable traits for survival of the plants in hot conditions. Therefore, amaranths remain highly competitive during hot growing seasons even in this Anthropocene era of higher carbon dioxide and hotter temperatures.

Capable of producing hundreds of thousands of seed per mother plant, *Amaranthus* species are rapidly evolving. They have variable genome sizes and shortened lifecycles, allowing for rapid adaptation to new environments (Das, 2016). Many wild species of *Amaranthus*, including some globally-significant weeds, are dioecious but most cultivated species are monoecious. In dioecious species to be an independently evolved trait (Neves et al., 2020), while extrachromosomal DNAs underpin rapid adaptive evolution, including herbicide resistance (Molin et al., 2020). Major weed amaranths (e.g. *A. retroflexus*, *A. spinosus, A. tuberculatus*) have become prominent pest problems world-wide (Heap, 2023) with expanding ranges in disturbed environments compared to natural ranges.

The weed species of *Amaranthus* are considered ubiquitous invaders of agricultural land, peri-urban fields and city lots. Many have developed resistance to commonly used herbicides of modern agriculture. Moghadam et al., in this Research Topic, discuss the genetic diversity of *A. retroflexus* collections in Spain, France and Iran, finding that multiple introductions of the weedy amaranth have caused distinct populations across this West Asian country that are now distinct from ones in Europe.

A series of grain-bearing *Amaranthus* species were domesticated by humans in the Andean and Mesoamerican zones of crop domestication. These include *A. caudatus*, *A. cruentus* and *A. hypochondriacus*, three species with complex inter-relationships, crop race structure and genomic divergence in the case of the former compared to the latter two. The research of Blair et al, presented in this Research Topic, discusses the diversity among and within the three cultivated grain species in relationship to potential progenitor and co-evolving bridge species of *A. hybridus* and the wild species, *A. powellii*. The wild-weedy *A. quitensis* was closely related to the Andean grain amaranth. This work describes a core collection for grain amaranths in the three cultivated species mentioned above.

Meanwhile in South Asia, a series of species were domesticated for their colorful and edible leaves as vegetable species (Das, 2016). These include *A. tricolor* and *A. viridis*. Wild relatives from Asia are *A. dubius* and *A. graecizans*. It is important to note that all grain amaranth species and some weedy types also produce edible leaves in young stages and these can be harvested as a vegetable crop before stalks and leaves get to tough. WorldVeg, as an agency concerned with nutritional status of undernourished populations around the world, has a dual-purpose breeding program for grain and leaf amaranths based in Tanzania.

While the versatility of the grain and vegetable has long been known, the uses of amaranth leaves in food industry is promising as well. The article by Howard et al. in this Research Topic, evaluates germplasm from the species discussed above, especially *A. cruentus*, as a source of betalain pigments which are more stable than anthocyanins for creating red or magenta colored juices and teas or other products with neutral or moderately acidic solutions. They suggest a system to harvest the stems and leaves for water-based extraction and find many existing varieties from seed companies, such as Hopi Red and Garnet to be potential sources for natural colorants.

Interest in grain, leaf vegetable and weed amaranths is expanding world wide and has been documented by a non-profit organization based out of Mexico and the United States called the Amaranth Institute. Of special interest is the potential improvements that various amaranth products provide to human nutrient status in various parts of the world (Myers, 2018).

The most-researched germplasm used by institute members have come from the United States Department of Agriculture (USDA) and some are geo-located with latitudinal and longitudinal coordinates. Many of the plant introductions trace back to a Rodale Research Institute program for collecting, selecting and crossing grain amaranths funded from 1976 to the mid-1990s, that was then handed over to the Gene Bank in Ames, Iowa, North Central Regional Plant Introduction center which is part of the Germplasm Resources Information Network. Fewer vegetable introductions are found in the Americas compared to Asia but many are at International Research or in commercial seed companies.

All the domesticated grain and vegetable amaranths contribute to diversified agriculture in multiple regions of the world. Historically, the grain amaranth species were centered around production in Mexico or Guatemala in the case of *A. cruentus* and *A. hypochondriacus*; and a few South American countries in the case of *A. caudatus* (Stetter et al., 2015). However, today the two Mexican species are an important food security crop in East Africa, where both leaves and seed are consumed. They have also become a new agronomic crop in China, Ukraine and the United States. Andean grain amaranths for the most part have not been used outside its region of origin in Bolivia, Ecuador and Peru but also have potential world-wide.

Vegetable amaranth types are grown traditionally in the Caribbean and East/South Asia. However, areas with large immigrant communities show demand for the leaves. The weed risk of many amaranths is well established and should be considered when moving seed of any species as they mix. Nevertheless, the papers presented herein demonstrate the broad and growing interest in grain and leaf amaranths and complement the first volume of our Research Topic (Riggins et al., 2021). The potential for crop transfers from one region to another justifies more research on all species and cultivars of amaranths, as exemplified by the research papers in this Research Topic. Interest is growing and potential for crop transfers from one region to another justify more research be done on all types of amaranths.

## Author contributions

MB: Project administration, Writing – original draft, Writing – review & editing. CR: Project administration, Writing – review & editing. AR: Project administration, Writing – review & editing.
